# Supplementary material for: Confounder-aware foundation modeling for accurate phenotype profiling in cell imaging
Source: Npj Imaging. 2025 Oct 22;3:52. doi: 10.1038/s44303-025-00116-9 (PMC12546604; doi:10.1038/s44303-025-00116-9)
Supplement: Supplementary file 1 — Supplementary Information [file 44303_2025_116_MOESM1_ESM.pdf]

Supplementary Information

Confounder-aware foundation modeling for accurate phenotype profiling in cell imaging

Giorgos Papanastasiou [1]*, Pedro P. Sanchez [2]*, Argyrios Christodoulidis [3], Guang Yang [4], Walter Hugo Lopez Pinaya [5]

Affiliations

[1] Artificial Intelligence, Data and Analytics Digital, Pfizer Inc, USA

[2] Sinkove, London, UK

[3] Research and Development Digital, Pfizer Inc, USA

[4] Bioengineering Department and Imperial-X, Imperial College London, London, UK

[5] Department of Biomedical Engineering, School of Biomedical Engineering & Imaging Sciences, King's College London, London, UK

* Sharing first co-authorship

Corresponding author: Dr Giorgos Papanastasiou: georgios.papanastasiou@pfizer.com

Supplementary Notes

Compound: Aloxistatin

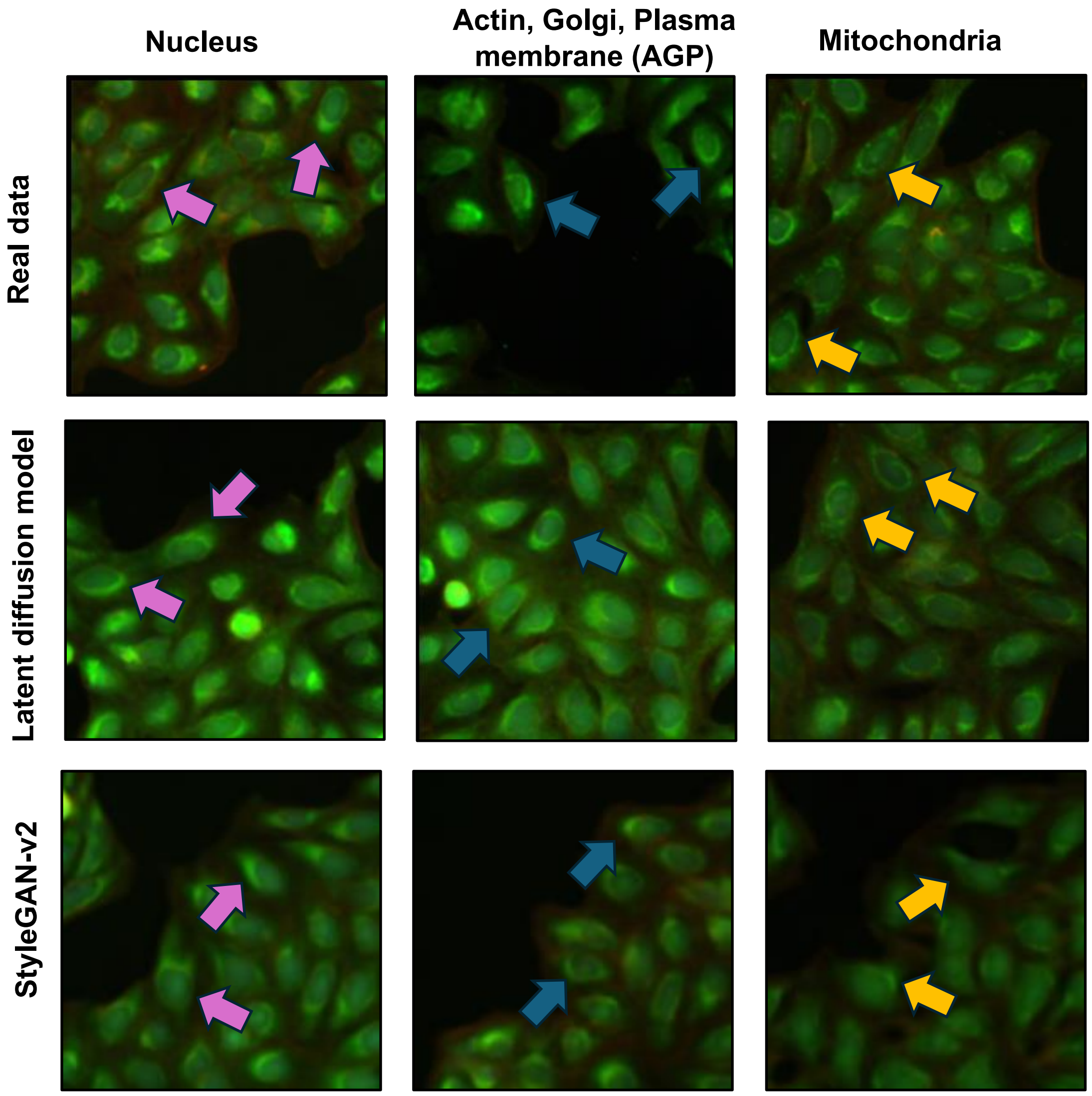

Supplementary Figure 1) Comparison of real and synthetic CP images across three channels (Nucleus; Actin, Golgi, Plasma membrane; Mitochondria), under the effect of a randomly selected compound (Aloxistatin). Representative examples are shown for real data, images synthesized by our diffusion model and StyleGAN-v2-generated images. While both generative models capture essential structural features, the diffusion model showcases enhanced visual realism and finer morphological details, especially in signal intensity, textures and organelle boundaries. These differences are noticeable across mostly the AGP and Mitochondria channels, demonstrating that the diffusion model preserves biological complexity and diversity more effectively (see magenta, blue, and orange arrows).

Compound: Imatinib

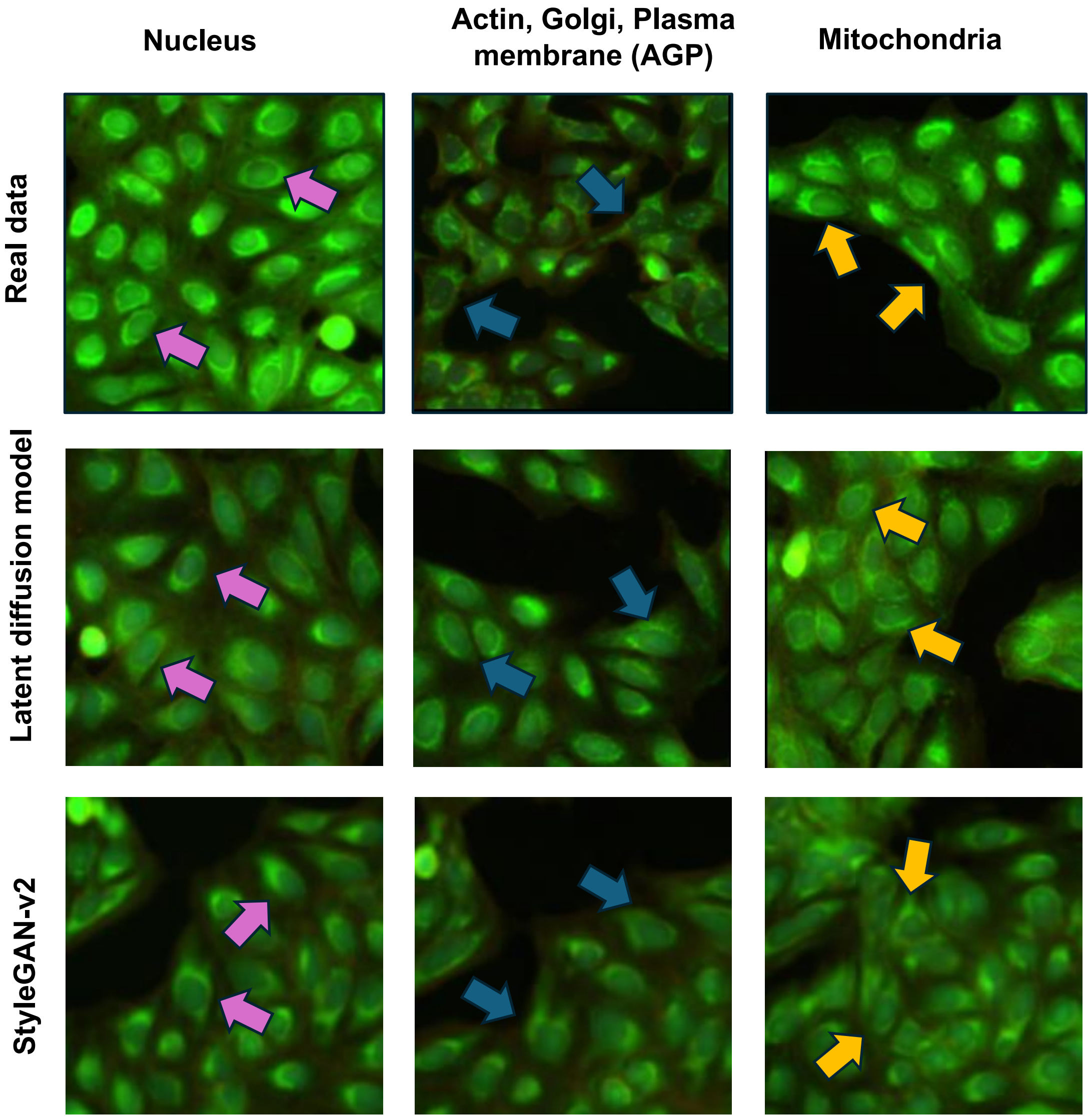

Supplementary Figure 2) Comparison of real and synthetic Cell Painting images across three channels (Nucleus, Actin, Golgi, Plasma membrane, and Mitochondria) under the effect of a randomly selected compound (Imatinib). Representative examples are shown for real data, images synthesized by our diffusion model and StyleGAN-v2-generated images. Both generative models capture key structural features. However, the diffusion model exhibits improved visual realism and finer morphological details, particularly in subtle textures, signal intensity and organelle boundaries. These differences are noticeable across mostly the AGP and Mitochondria channels, with the diffusion model better preserving biological complexity and diversity (see magenta, blue, and orange arrows).

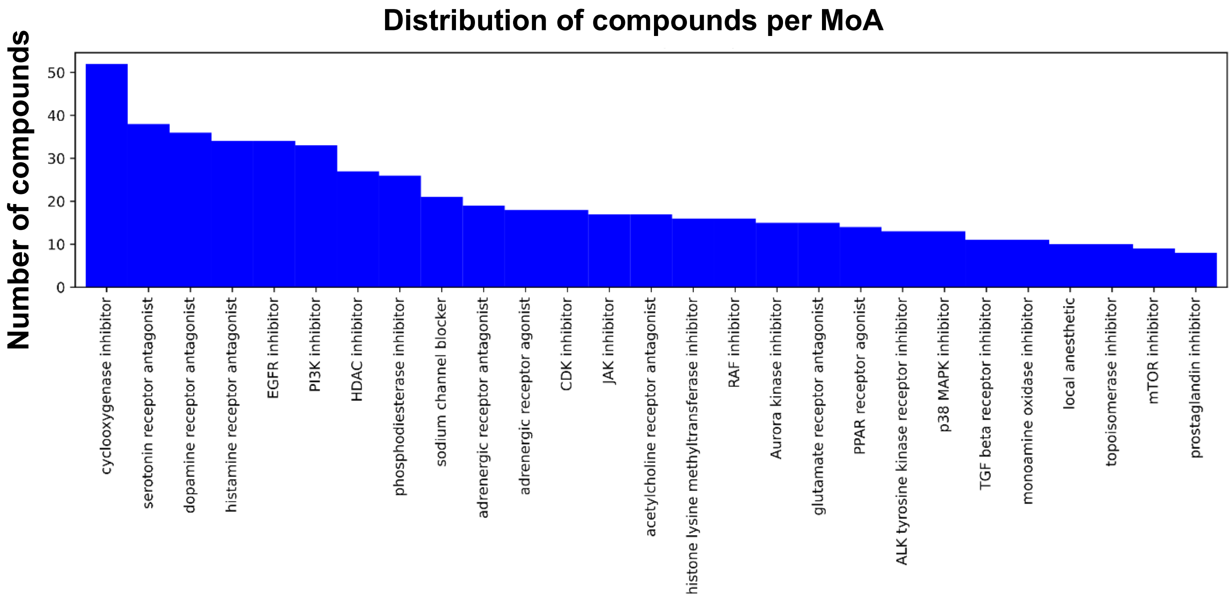

a)

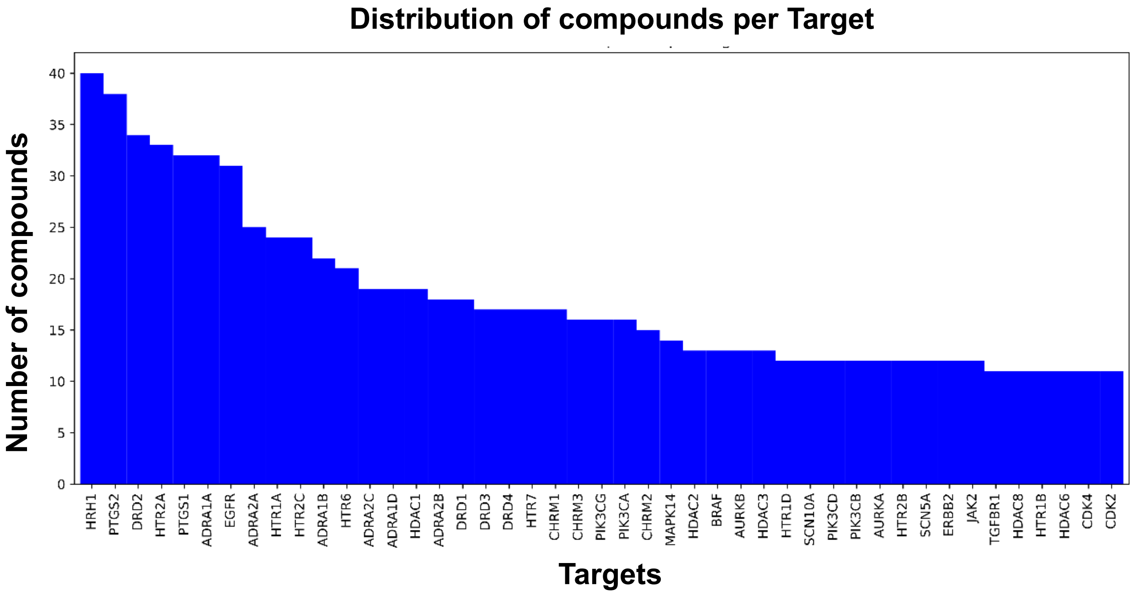

b)

Supplementary Figure 3) Distribution of compounds across MoAs (a) and across the first 45 selected compound targets (b), ranked by compound numbers, from the Broad Institute Drug Repurposing Hub. MoA: mechanism of action.

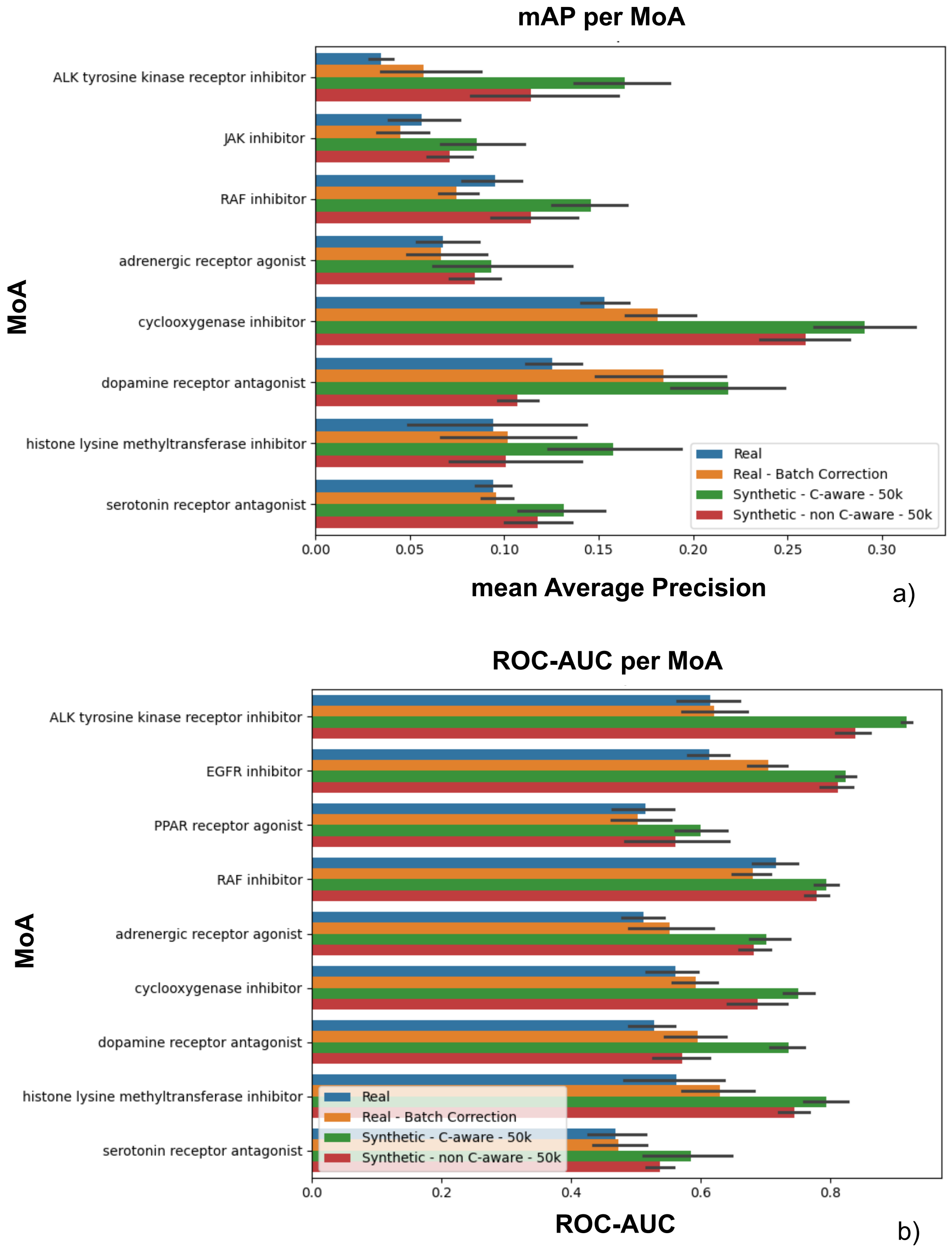

Supplementary Figure 4) mAP (mean average precision) (a) and ROC-AUC (b) per MoA, highlighting instances where the confounder-aware model outperformed other approaches.

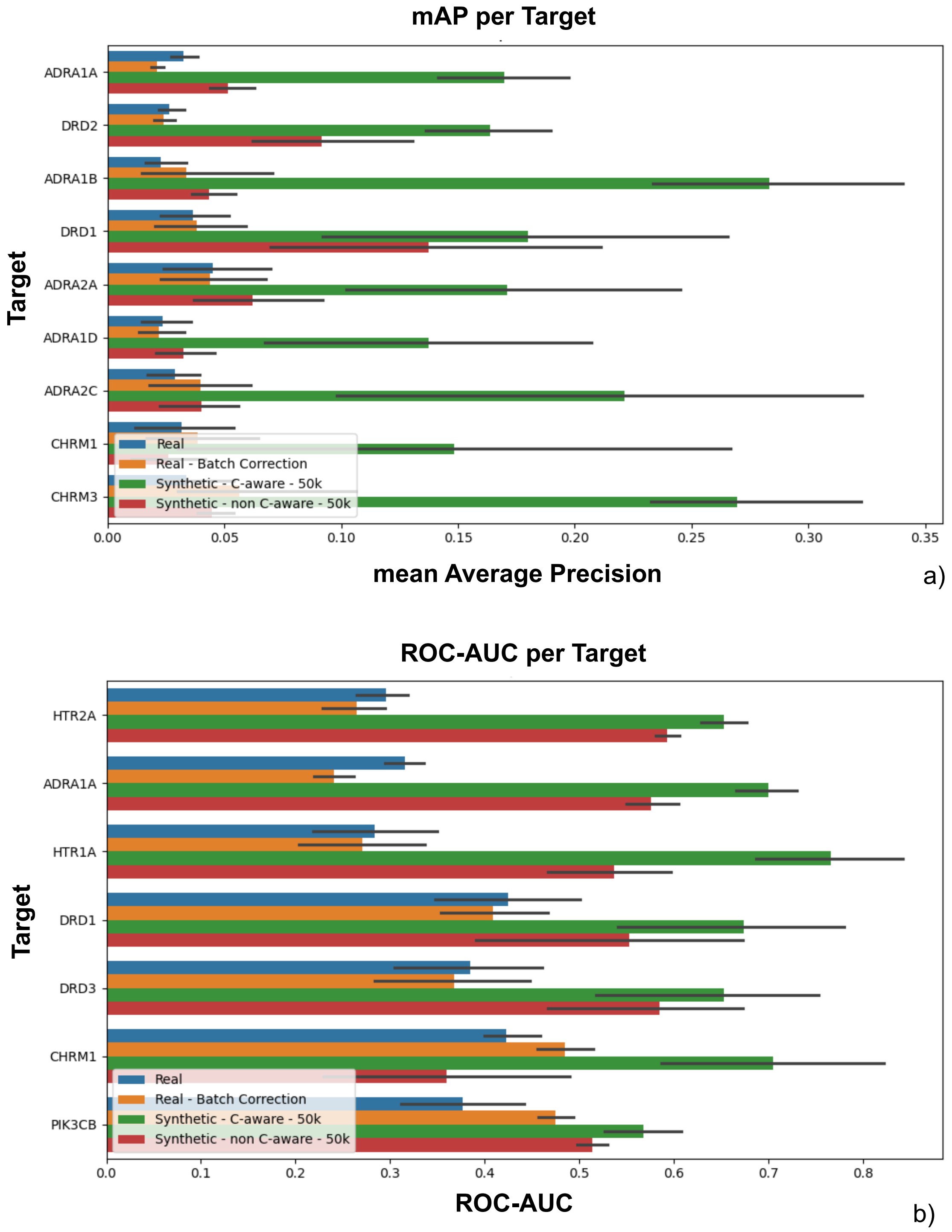

Supplementary Figure 5) mAP (mean average precision) and ROC-AUC per compound target, highlighting instances where the confounder-aware model outperformed other approaches.

Supplementary Table 1) Quantitative comparison between our latent diffusion model and a StyleGAN-v2 baseline [16] trained under matched conditions (9 most prevalent chemical compounds). Metrics were computed using Inception-v3 features on 9,000 generated images (1,000 per compound). Diffusion model outperforms StyleGAN-v2 across all fidelity and diversity metrics.

**Model**	**FID ↓**	**Precision ↑**	**Recall ↑**	**Density ↑**	**Coverage ↑**
Latent Diffusion Model (Ours)	17.3	0.77	0.62	0.83	0.78
StyleGAN-v2 (GAN) [16]	47.8	0.40	0.02	0.19	0.24

Supplementary Table 2) Silhouette scores and within-cluster variance were calculated using the same batches and cell profiles employed in the UMAP visualizations. Results are presented for different data sources and averaged over five random seeds. Mean and standard deviation (SD) are reported. Silhouette scores were computed using actual batch labels to quantify how well batch effects are separated in UMAP space. Higher values indicate clearer batch separation, while negative values suggest overlapping or poorly defined batch structure. The within-cluster variance of compounds metric was derived by applying HDBSCAN clustering to UMAP embeddings and computing the variance in the number of unique compounds per cluster (normalized using min-max scaling). This metric captures how well compound-specific effects are separated within each cluster. All metrics were computed across five random seeds to ensure robustness. The confounder-aware foundation model consistently outperforms other approaches, showing both improved batch disentanglement and clearer compound separation. Note that as the non-confounder-aware model is batch-agnostic, it is not possible to create silhouette scores based on batch labels.

Data source	Seed 1 (42)	Seed 2 (1025)	Seed 3 (2)	Seed 4 (164211)	Seed 5 (901234)	Mean (SD)
**Silhouette scores**						
**Confounder - aware FM data**	0.34	0.33	0.33	0.33	0.33	0.33 (0.00)
**Non-Confounder - aware FM data**	-	-	-	-	-	-
**Real data**	-0.25	-0.25	-0.24	-0.27	-0.25	-0.25 (0.00)
**Harmony Batch-Corrected data**	-0.29	-0.29	-0.26	-0.26	-0.28	-0.28 (0.00)
**Within-cluster variance of compounds**						
**Confounder - aware FM data**	0.14	0.14	0.13	0.14	0.14	0.14 (0.00)
**Non-Confounder - aware FM data**	0.08	0.10	0.08	0.06	0.06	0.08 (0.01)
**Real data**	0.02	0.02	0.02	0.03	0.02	0.02 (0.00)
**Harmony Batch-Corrected data**	0.01	0.02	0.02	0.01	0.01	0.01 (0.00)

Supplementary Table 3) Number of compounds used for MoA and target prediction, for each fold and task.

Task	Fold	Used for evaluation	Used for reference "subprofiles"	Total	Used to evaluate seen compounds	Used to evaluate unseen compounds
MoA	1	395	130	525	281	114
MoA	2	395	130	525	280	115
MoA	3	395	130	525	278	117
MoA	4	395	130	525	278	117
MoA	5	395	130	525	279	116
MoA	6	395	130	525	273	122
MoA	7	395	130	525	277	118
MoA	8	395	130	525	284	111
MoA	9	395	130	525	278	117
MoA	10	395	130	525	281	114
Target	1	368	97	465	229	139
Target	2	373	92	465	234	139
Target	3	370	95	465	231	139
Target	4	368	97	465	229	139
Target	5	369	96	465	230	139
Target	6	367	98	465	228	139
Target	7	370	95	465	231	139
Target	8	369	96	465	230	139
Target	9	367	98	465	228	139
Target	10	377	88	465	238	139

Supplementary Table 4) Label-shuffling control experiment for MoA and target prediction tasks. This table reports mean and standard deviation (std) of ROC-AUC scores for both standard and label-shuffled evaluations. Shuffling the sample-label mapping consistently reduced performance to chance levels (ROC-AUC ≈ 0.49–0.51), confirming that the models rely on genuine biological signal rather than spurious correlations. Results are shown for real, Harmony batch-corrected, and synthetic datasets, across confounder-aware and non-confounder-aware foundation models, including evaluations on unseen data.

**MoA prediction**				
**Dataset**	**Standard AUCROC mean**	**Standard AUCROC std**	**Shuffled AUCROC mean**	**Shuffled AUCROC std**
Real	0.59	0.02	0.49	0.02
Harmony Batch Corrected	0.61	0.02	0.51	0.02
Confounder-aware FM - 5k	0.63	0.02	0.49	0.01
Confounder-aware FM - 50k	0.66	0.02	0.51	0.01
Non-Confounder-aware FM - 5k	0.64	0.02	0.49	0.01
Non-Confounder-aware FM - 50k	0.64	0.02	0.50	0.03
Unseen - Real	0.57	0.02	0.51	0.01
Unseen - Harmony Batch Corrected	0.58	0.02	0.50	0.02
Unseen - Synthetic - C-aware - 5k	0.62	0.02	0.50	0.01
Unseen - Synthetic - C-aware - 50k	0.65	0.02	0.51	0.01
Unseen - Non-Confounder-aware FM - 5k	0.65	0.03	0.49	0.02
Unseen - Non-Confounder-aware FM - 50k	0.66	0.02	0.51	0.02
**Target prediction**				
**dataset**	**mean AUROC**	**std AUCROC**	**Shuffled mean AUCROC**	**Shuffled std AUCROC**
Real	0.38	0.02	0.51	0.02
Harmony Batch Corrected	0.37	0.02	0.51	0.01
Confounder-aware FM - 5k	0.62	0.02	0.49	0.03
Confounder-aware FM - 50k	0.66	0.02	0.51	0.02
Non-Confounder-aware FM - 5k	0.57	0.02	0.49	0.03
Non-Confounder-aware FM - 50k	0.59	0.02	0.50	0.03
Unseen - Real	0.45	0.01	0.50	0.02
Unseen - Harmony Batch Corrected	0.41	0.01	0.50	0.03
Unseen - Synthetic - C-aware - 5k	0.64	0.02	0.51	0.03
Unseen - Synthetic - C-aware - 50k	0.73	0.02	0.51	0.02
Unseen - Non-Confounder-aware FM - 5k	0.54	0.01	0.48	0.02
Unseen - Non-Confounder-aware FM - 50k	0.59	0.02	0.51	0.03
